# Room-temperature synthesis of zinc oxide nanoparticles in different media and their application in cyanide photodegradation

**DOI:** 10.1186/1556-276X-8-516

**Published:** 2013-12-06

**Authors:** Abdulaziz Bagabas, Ahmad Alshammari, Mohamed FA Aboud, Hendrik Kosslick

**Affiliations:** 1 Petrochemicals Research Institute, King Abdulaziz City for Science and Technology (KACST), P.O. Box 6086, Riyadh 11442, Saudi Arabia; 2 Sustainable Energy Technologies (SET) Center, College of Engineering, King Saudi University, P.O. BOX 800, Riyadh 11421, Saudi Arabia; 3 Institute of Chemistry, University of Rostock, Albert-Einstein-Strasse 3a, Rostock D-18059, Germany

**Keywords:** Room-temperature synthesis, Zinc oxide nanoparticles, Cyanide photodegradation

## Abstract

Cyanide is an extreme hazard and extensively found in the wastes of refinery, coke plant, and metal plating industries. A simple, fast, cost-effective, room-temperature wet chemical route, based on cyclohexylamine, for synthesizing zinc oxide nanoparticles in aqueous and enthanolic media was established and tested for the photodegradation of cyanide ions. Particles of polyhedra morphology were obtained for zinc oxide, prepared in ethanol (ZnO_E_), while spherical and some chunky particles were observed for zinc oxide, prepared in water (ZnO_W_). The morphology was crucial in enhancing the cyanide ion photocatalytic degradation efficiency of ZnO_E_ by a factor of 1.5 in comparison to the efficiency of ZnO_W_ at an equivalent concentration of 0.02 wt.% ZnO. Increasing the concentration wt.% of ZnO_E_ from 0.01 to 0.09 led to an increase in the photocatalytic degradation efficiency from 85% to almost 100% after 180 min and a doubling of the first-order rate constant (*k*).

## Background

Cyanide has numerous applications in industry such as chelating agent, electroplating, pharmaceuticals, and mining [1,2]. This extensive use of cyanide results in the generation of a huge amount of cyanide waste and increases the cyanide spill risk to the environment [3,4]. Thus, cyanide must be treated before discharging. Different protocols such as adsorption, complexation, and oxidation are used for abating cyanides [1,2,5-7]. The procedures other than oxidation give highly concentrated products in which toxic cyanides still exist [8,9].

Highly powerful, economically method is the photocatalytic oxidation of cyanide, which has been demonstrated in several studies [10-17]. However, an inexpensive photocatalyst is needed for the economical removal of large quantities of cyanide. ZnO is one of the most promising materials for executing this task, as an alternative to the widely used, relatively expensive titania (TiO_2_). Although researchers recognized comparable photodegradation mechanisms with both ZnO and TiO_2_, they proved that ZnO was the superior photocatalyst in degrading pesticide carbetamide, herbicide triclopyr, pulp mill bleaching wastewater, 2-phenylphenol, phenol, blue 19, and acid red 14. This superiority of ZnO photocatalytic activity is because it has more active sites, higher reaction rates, and is more effective in generating hydrogen peroxide [18].

Due to its direct, wide bandgap of 3.37 eV, ZnO has a wide range of applications in optoelectronic devices [19] such as light-emitting diodes, photodetectors, and p-n homojunctions. The large exciton binding energy of 60 meV [19], compared to that of GaN (approximately 25 meV) [20], enhances the luminescence efficiency of the emitted light even at room temperature and higher. The visible photoluminescence (PL) emission at approximately 2.5 eV (approximately 495 nm), originated from intrinsic defects [21], makes ZnO suitable for applications in field emission and vacuum fluorescent displays.

Many techniques including chemical vapor deposition [22], pulsed laser deposition [23], molecular beam epitaxy [24], sputtering [25], hydrothermal synthesis [26], and oxidation of metallic zinc powder [27,28] have been used to prepare ZnO in different forms and structures for various applications. Nanoparticulate form enhances the catalytic activity due to its large surface area and the presence of vacancies and uncoordinated atoms at corners and edges. The photocatalytic activity is also improved by bandgap engineering, as a result of the quantum confinement effect [29-31].

A well-controlled synthesis process at room temperature is needed for the economical use of ZnO in catalytic applications such as water treatment and other environmental applications. Herein, we are reporting, for the first time to the best of our knowledge, a direct, simple, room-temperature synthesis method for ZnO nanoparticles using cyclohexylamine (CHA), as a precipitating agent, and zinc nitrate hexahydrate, as a source of zinc, in both aqueous and ethanolic media. The synthesized ZnO nanoparticles were examined as a photocatalyst for the degradation of the highly toxic cyanide anion [CN^-^_(aq)_] in the aqueous medium at room temperature. The kinetics for cyanide photodegradation were investigated with respect to ZnO concentration of weight percentage.

## Method

### Materials

Zinc nitrate hexahydrate (pure, POCH), cyclohexylamine (GC >99%, Merck, Whitehouse Station, NJ, USA), absolute ethanol (EtOH, 99.9%, Scharlau, Sentmenat, Barcelona, Spain), potassium cyanide (≥97%, Sigma-Aldrich, St. Louis, MO, USA), potassium iodide (≥99.5%, Sigma-Aldrich), and ammonia solution (28-30% NH_3_ basis, Sigma-Aldrich) were commercially available and were used as received. Deionized water (18.2 MΩ.cm)was obtained from a Milli-Q water purification system (Millipore, Billerica, MA, USA).

### Synthesis of ZnO nanoparticles in water (ZnO_W_) and in ethanol (ZnO_E_)

Thirty millimoles of zinc nitrate hexahydrate was dissolved in 60 ml of water at room temperature, under continuous magnetic stirring. In a separate beaker, 60 mmol of CHA was dissolved in 20 ml water at room temperature. The CHA solution was poured into the zinc solution, resulting in a white precipitate upon magnetic stirring. An extra amount of 80 ml water was added to the reaction mixture, which was left stirring for 4 days. The precipitate was filtered off through an F-size fritted filter and then was washed with 100 ml water. The precipitate was dried at room temperature under vacuum for 1 day. After drying, the precipitate was mixed with 300 ml water and was magnetically stirred for 1 day for the removal of any impurity. The precipitate was filtered off and was dried room temperature under vacuum to give 2.43 g (yield% = 89.7). This dried sample was then calcined at 500°C under air for 3 h. The temperature was ramped from room temperature to the target temperature by 1°C/min. Inductively coupled plasma (ICP) elemental analysis was carried out for the uncalcined sample, which proved the formation of zinc oxide at room temperature with a formula of ZnO **·** 1/2H_2_O [Zn (cal. 72.3%, exp. 72.9%)].

In addition, the same procedure was carried out to prepare ZnO nanoparticles in ethanolic medium instead of water. The precipitate gave 2.572 g (yield% = 98.1) of ZnO **·** 1/3H_2_O, as proven by ICP elemental analysis [Zn (cal. 74.8%, exp. 74.2%)]. Both of uncalcined ZnO nanoparticles in water (ZnO_W_) and in ethanol (ZnO_E_) were found to be soluble in HCl and NaOH, evidencing the chemical identity of ZnO.

### Material characterization

Inductively coupled plasma (ICP) was used to determine the percentage of the zinc component in uncalcined ZnO samples, obtained at room temperature. Brunauer, Emmett, and Teller surface areas (BET-SA) and pore size distribution of the catalysts were obtained on Micrometrics Gemini III-2375 (Norcross, GA, USA) instrument by N_2_ physisorption at 77 K. Prior to the measurements, the known amount of the catalyst was evacuated for 2 h at 150°C. Diffuse reflectance infrared Fourier transform (DRIFT) spectra of ground, uncalcined ZnO powder samples, diluted with IR-grade potassium bromide (KBr), were recorded on a Perkin Elmer FTIR system spectrum GX (Waltham, MA, USA) in the range of 400 to 4,000 cm^-1^ at room temperature. X-ray diffraction (XRD) patterns were recorded for phase analysis and crystallite size measurement on a Philips X pert pro diffractometer (Eindhoven, Netherlands), operated at 40 mA and 40 kV by using CuK_α_ radiation and a nickel filter, in the 2-theta range from 2° to 80° in steps of 0.02°, with a sampling time of 1 s per step. The crystallite size was estimated using Scherer's equation. XRD patterns were recorded for uncalcined and calcined (500°C) ZnO materials. The morphology was investigated using a field-emission scanning electron microscope (FE-SEM model: FEI-200NNL, Hillsboro, OR, USA), equipped with an energy-dispersive X-ray (EDX) spectrometer for elemental analysis, and a high-resolution transmission electron microscope (HRTEM model: JEM-2100 F JEOL, Akishima-shi, Tokyo, Japan). Carbon-coated copper grids were used for mounting the samples for HRTEM analysis. Solid-state ultraviolet-visible (UV-vis) absorption spectra for calcined ZnO powder samples were recorded on a Perkin Elmer Lambda 950 UV/Vis/NIR spectrophotometer, equipped with a 150-mm snap-in integrating sphere for capturing diffuse and specular reflectance.

### Photocatalytic test

The photocatalytic evaluation was carried out using a horizontal cylinder annular batch reactor. A black light-blue florescent bulb (F18W-BLB) was positioned at the axis of the reactor to supply UV illumination. Reaction suspension was irradiated by UV light of 365 nm at a power of 18 W. The experiments were performed by suspending 0.01, 0.02, 0.03, 0.05, 0.07, or 0.09 wt.% of calcined ZnO into a 300-ml, 100 ppm potassium cyanide (KCN) solution, with its pH adjusted to 8.5 by ammonia solution. The reaction was carried out isothermally at 25°C, and samples of the reaction mixture were taken at different intervals for a total reaction time of 360 min. The CN^-^_(aq)_ concentration in the samples was estimated by volumetric titration with AgNO_3_, using potassium iodide to determine the titration end-point [32]. The percentage of degradation of CN^-^_(aq)_ has been measured by applying the following equation: %Degradation = (C_o_ - C)/C_o_ × 100, where C_o_ is the initial concentration of CN^-^_(aq)_ and C is the concentration of uncomplexed CN^-^_(aq)_ in solution.

## Results and discussion

### Formation of ZnO nanoparticles in an aqueous and ethanolic media

Formation of zinc oxide from the combination of zinc nitrate hexahydrate and CHA either in aqueous or ethanolic medium can be illustrated by Equation 1:

(1) $$\begin{array}{l} \mathrm{Zn}{{\left({\mathrm{NO}}_{3}\right)}_{2}}_{\left(\mathrm{aq}\;\mathrm{or}\;\mathrm{alc}\right)}+2C_{6}H_{11}{\mathrm{NH}}_{2\left(\mathrm{aq}\;\mathrm{or}\;\mathrm{alc}\right)}\\ \;\;+H_{2}O_{\left(l\right)}\to {\mathrm{ZnO}}_{\left(\mathrm{nc}\right)}\\ \;\;+2C_{6}H_{11}{\mathrm{NH}}_{3}{{\mathrm{NO}}_{3}}_{\left(\mathrm{aq}\;\mathrm{or}\;\mathrm{alc}\right)} \end{array}$$

CHA, according to Equation 1, acts as a base in the Brønsted-Lowry sense, but not as a base in the Lewis sense (a ligand). This behavior of CHA was proven by the isolation and determination of the structure of cyclohexylammonium nitrate crystals by single-crystal XRD [33]. This observed Brønsted-Lowry activity of CHA can be attributed to its moderate base strength (pK_b_ = 3.36) when hydrolyzing in water according to Equation 2:

(2) $$C_{6}H_{11}{\mathrm{NH}}_{2\left(\mathrm{aq}\right)}+H_{2}O_{\left(l\right)}⇄C_{6}H_{11}{\mathrm{NH}}_{3}{{}^{+}}_{\left(\mathrm{aq}\right)}+{\mathrm{OH}}_{\left(\mathrm{aq}\right)}$$

Due to the high basicity of the CHA solution (pH = 12.5), zinc ions react with the hydroxide ions and form different hydroxyl complexes such as [ZnOH]^+^, [Zn(OH)_2_]_(aq)_, [Zn(OH)_3_]^-^_(aq)_, and [Zn(OH)_4_]^2-^_(aq)_. Furthermore, the high basicity makes the chemical potential of hydroxide ion [OH]^-^ high, leading to a shift in the equilibrium in Equation 3 toward the formation of oxide ion (O^2-^):

(3) $${{2\mathrm{OH}}^{-}}_{\left(\mathrm{aq}\right)}\;⇄{O^{2-}}_{\left(\mathrm{aq}\right)}+H_{2}O_{\left(l\right)}$$

The formation of zinc hydroxide complexes and oxide ions shifts the equilibrium in Equation 2 forward, causing further protonation of CHA and the formation of more hydroxide ions.

The formation of oxide ion according to Equation 3 is responsible for the construction of Zn-O-Zn bonds by transforming the zinc hydroxide complexes into solid-phase according to Equation 4:

(4) $$\begin{array}{l} 2{{\left[\mathrm{Zn}{\left(\mathrm{OH}\right)}_{n}\right]}^{2-n}}_{\left(\mathrm{aq}\right)}\;⇄{{\left[{\mathrm{Zn}}_{2}O{\left(\mathrm{OH}\right)}_{2n-2}\right]}^{4-2n}}_{\left(\mathrm{aq}\right)}\\ \;\;+H_{2}O_{\left(l\right)} \end{array}$$

Equation 4 shows that the construction of ZnO crystal takes place via the interaction between the surface hydroxide of the growing crystals and the hydroxide ligands of the zinc complexes. Therefore, the formation of ZnO, according to the above proposed mechanism, is due to the high basicity of the reaction medium, which causes an increase in the concentration of the precursors (zinc hydroxide complexes) and an increase in the chemical potential of hydroxide ions [34].

### BET surface area

In general, specific surface area is a significant microstructural parameter of materials particles, which depends on the geometrical shape and porosity. It is also well known that a large surface area could be an important factor, prompting the photocatalytic degradation of organic materials [35]. The specific surface areas and pore volumes of our ZnO, prepared in either EtOH or H_2_O medium, are presented in Table 1. It is clear from the table that the BET surface area and pore volumes are observed to change marginally by changing the reaction medium. Interestingly, our results showed that in comparison with the morphology of ZnO nanoparticles, the surface area is not a significant parameter in photocatalytic activity; ZnO prepared in ethanol with higher efficiency (see Table 1) has somewhat lower surface area (7.51 m^2^/g) in comparison with ZnO prepared in H_2_O (12.41 m^2^/g). Lower photocatalytic activity of ZnO prepared in H_2_O can be attributed to the shape and morphology as we will discuss on details later on.

**Table 1:** **BET surface area and pore volume of calcined ZnO nanoparticles, prepared either in EtOH or H**_**2**_**O**

**Sample**	**BET-SA (m**^**2**^**/g)**	**Pore volume (cm**^**3**^**/g)**
ZnO_E_	7.51	0.02
ZnO_W_	12.41	0.05

### DRIFT investigation

**Figure 1:**
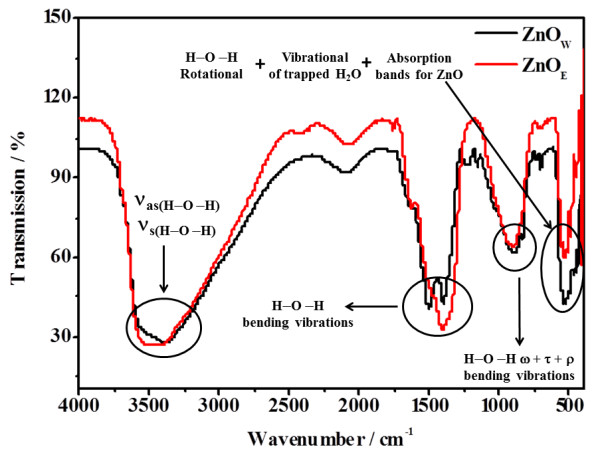
**DRIFT spectra of uncalcined ZnO nanoparticles, prepared either in EtOH (ZnO**_**E**_**) or H**_**2**_**O.**

Figure 1 shows the DRIFT spectra of the uncalcined ZnO nanoparticles, prepared in either H_2_O or EtOH medium. The absorption bands in the region of 600 to 400 cm^-1^ include those for crystal (lattice) and coordinated water as well as ZnO. The absorption bands for ZnO are weak and overlap with those of rotational H-O-H vibration and vibrational of trapped H_2_O. The asymmetric and symmetric stretching H-O-H vibration bands are observed between 3,600 and 3,200 cm^-1^, while the bending H-O-H vibration bands are observed between 1,630 and 1,600 cm^-1^[36,37]. The doublet band at approximately 1,400 cm^-1^ can be ascribed to H-O-H bending vibrations. The bands, observed between 880 and 650 cm^-1^, can be attributed to the bending vibrational modes (wagging, twisting, and rocking) of coordinated water molecules. The water diagnosis by DRIFT is in agreement with the ICP-prediction of water presence in the uncalcined ZnO_W_ and ZnO_E_ samples (see synthesis in the ‘Method’ section).

### XRD investigation

**Figure 2:**
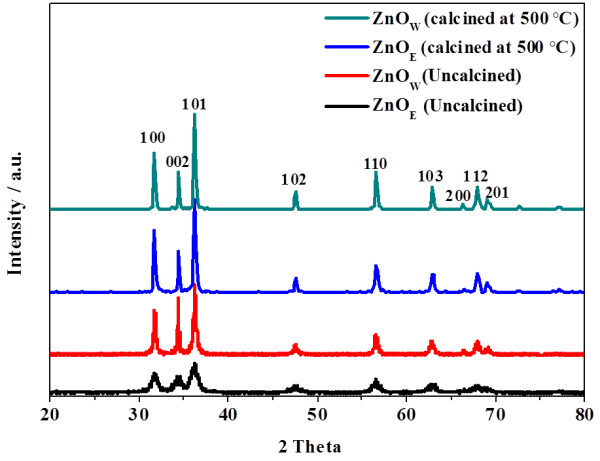
**XRD patterns of uncalcined and calcined (500°C) ZnO nanoparticles, prepared in H**_**2**_**O (ZnO**_**W**_**) and EtOH (ZnO**_**E**_**).**

Figure 2 shows the XRD diffraction patterns of uncalcined and calcined ZnO nanoparticles, prepared in water and ethanol. The patterns consist of broad peaks, which match the common ZnO hexagonal phase, i.e., wurtzite structure [80–0074, JCPDS]. The sharper and higher peak intensities of the uncalcined ZnO_W_ than those of the uncalcined ZnO_E_ imply that the latter has a smaller crystallite size than that of the former. The average crystallite size, estimated by Scherrer's equation for the (100), (002), and (101) diffraction peaks, for the uncalcined ZnO_E_ is almost half that of the uncalcined ZnO_W_ (Table 2). After calcination, however, both ZnO_E_ and ZnO_W_ had the same average crystallite size of 28.8 nm (Table 2). Such observation could be attributed to the difference in the number of moles of water of crystallization in each material, resulting in more shrinkage relative to the particle coarsening effect upon calcination for the ZnO_W_[38].

**Table 2:** **Average crystallite size of uncalcined [a] and calcined [b] ZnO**_**E**_**and ZnO**_**W**_

**Miller indices (*hkl*)**				**Average crystallite size (nm)**
	**100**	**002**	**101**	
ZnO_E_^a^	13.9	14.5	18.2	15.6
ZnO_W_^a^	33.5	28.9	39.3	33.9
ZnO_E_^b^	33.5	24.8	28.2	28.8
ZnO_W_^b^	33.5	24.8	28.2	28.8

^a^Uncalcined ZnO_E_ and ZnO_W_; ^b^calcined ZnO_E_ and ZnO_W_.

### SEM investigation

**Figure 3:**
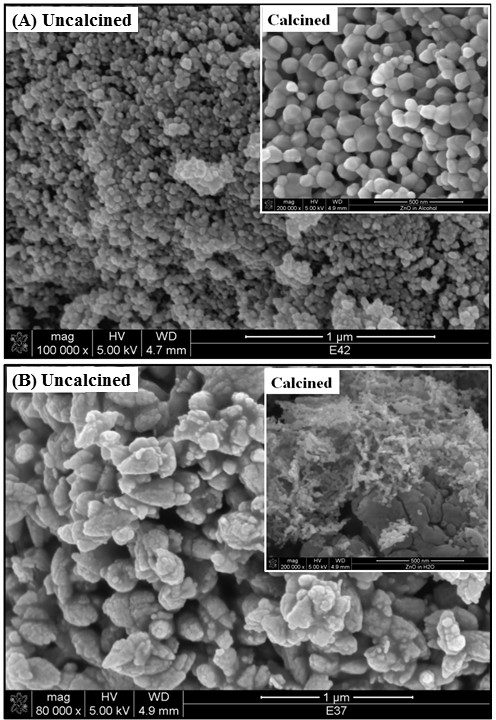
**SEM of uncalcined and calcined ZnO nanoparticles, prepared either in EtOH (ZnO**_**E**_**) (A) or H**_**2**_**O (ZnO**_**W**_**) (B).**

Figure 3A shows the SEM images of uncalcined and calcined (inset) ZnO_E_ samples, while Figure 3B shows the SEM images of uncalcined and calcined (inset) ZnO_W_ samples. Uncalcined ZnO_E_ sample is composed of homogeneously defined nanoparticles. On the other hand, uncalcined ZnO_W_ sample is made of irregularly shaped, overlapped nanoparticles. Removal of lattice water upon calcination process enhanced the nanoparticles' features. Regular, polyhedral nanoparticles were observed for ZnO_E_ after calcination. Inhomogeneous, spherical particles along with some chunky particles were observed for ZnO_W_. The EDX analyses (not shown here) for uncalcined and calcined samples indicate the purity of all the synthesized samples with no peaks other than Zn and O.

### TEM investigation

**Figure 4:**
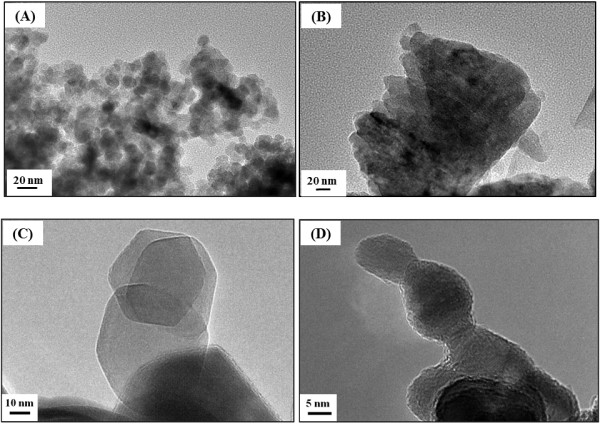
**TEM images of the uncalcined ZnO**_**E **_**(A) and ZnO**_**W **_**(B), and calcined ZnO**_**E **_**(C) and ZnO**_**W **_**(D).**

TEM images (Figure 4) of un- and calcined ZnO samples supported the SEM micrographs in confirming the morphology of ZnO nanoparticles. Un- and calcined ZnO_E_ nanoparticles adopt hexagonal shape, which is consistent with the regular, polyhedral morphology observed by SEM (Figure 3A, inset), with an average particle size of approximately 40 nm, obtained from TEM (Figure 4C). However, calcined ZnO_W_ nanoparticles adopt irregular spherical shape with an average particle size of approximately 15 nm (Figure 4D), which is consistent with the observed morphology by SEM (Figure 3B, inset). The more uniform polyhedral particles of ZnO_E_ could be attributed to the lower polarity of ethanol, compared to that of water, leading to slower ionization and deposition rate [39] and inhomogeneous nucleation that favor the polyhedral-shaped particles [40].

**Figure 5:**
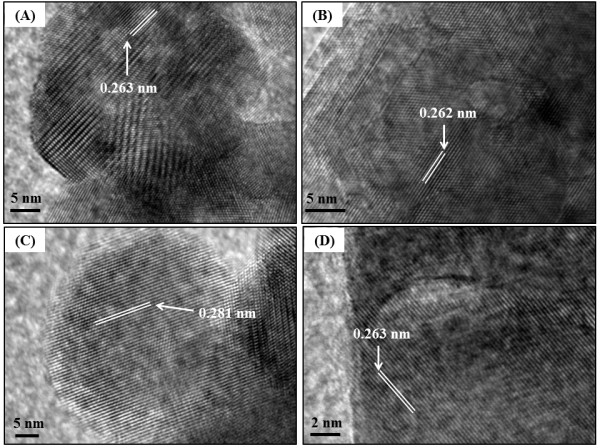
**HRTEM images of the uncalcined ZnO**_**E **_**(A) and ZnO**_**W **_**(B), and calcined ZnO**_**E **_**(C) and ZnO**_**W **_**(D).**

In order to study deeply shape and crystallinity of ZnO nanoparticles, prepared in ethanol and water, and further to confirm the XRD patterns, high-resolution TEM (HRTEM) was performed. This technique has provided us information regarding the nature of the crystal faces. HRTEM images of un- and calcined ZnO_E_ and ZnO_W_ are shown in Figure 5A, B, C, D. These images obviously confirmed that un- and calcined ZnO (Figure 5A, C) prepared in ethanol has hexagonal shape, whereas irregular spherical shape of ZnO prepared in water (Figure 5B, D). In addition, from HRTEM images of un- and calcined ZnO prepared ethanol and water, one can clearly observe the crystal planes of ZnO. The lattice plane fringes of the ZnO nanoparticles are used to calculate the d-spacing values, and they were compared with those of bulk ZnO (the values in Table 3), indicating the formation of ZnO nanocrystals with different morphology depending on the reaction medium. From Table 3, the distances between the two lattice planes for un- and calcined ZnO_E_ were around 0.263 and 0.281 nm, which correspond to the d-spacing of the (002) and (100) crystal planes, respectively, of the wurtzite ZnO. On another hand, the interplanar spacings of un- and calcined ZnO_W_ were around 0.262 and 0.263, corresponding well to the (002) planes of ZnO.

**Table 3:** **The inter planar spacing and diffraction planes of un- and calcined ZnO**_**E**_**and ZnO**_**W**_

**Samples**	**d-spacing calculated from HRTEM (nm)**	**d-spacing in bulk ZnO (nm)**	**Miller indices (*hkl*) assignment**
ZnO_E_ (uncalcined)^a^	0.263	0.260	002
ZnO_W_ (uncalcined)^b^	0.262	0.260	002
ZnO_E_ (calcined)^c^	0.281	0.281	100
ZnO_W_ (calcined)^d^	0.263	0.260	002

^a^Figure 5A; ^b^Figure 5B; ^c^Figure 5C; ^d^Figure 5D.

### UV-vis investigation

**Figure 6:**
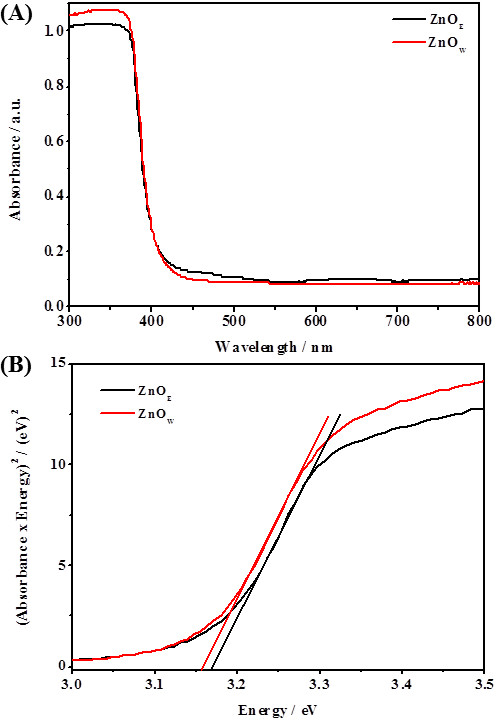
**UV-vis absorption spectrum (A) and direct bandgap (B) for calcined ZnOw and ZnO**_**E**_**, respectively.**

Figure 6A exhibits the UV-vis absorption spectra for the calcined ZnO_E_ and ZnO_W_ samples. The ZnO_E_ sample showed slightly less absorbance between 300 and 400 nm than ZnO_W_. This decrease in absorbance could be attributed to the larger particle size of ZnO_E_, which in turn increases its Rayleigh scattering [41]. The direct bandgap (*E*_*g*_) estimations from these spectra for ZnO_E_ and ZnO_W_ are depicted in Figure 6B, where the *x*-axis is the photon energy (*E*) in electron-volt (eV) and *y*-axis is the square of the product of absorbance (*A*) and energy (*AE*)^2^. The *E*_*g*_ for ZnO_E_ was 3.17 eV, while that for ZnO_W_ was 3.16 eV. Such observation implies that the optical properties of these materials are not affected by the synthesis medium.

### Photocatalytic degradation of cyanide

#### Synthesis medium effect on photocatalytic oxidation

The mechanism for the photocatalytic oxidation of cyanide by zinc oxide can be illustrated as follows [41]:

$$\begin{array}{l} \mathrm{ZnO}+2hv=\mathrm{ZnO}\;\left(2h^{+}+2e^{-}\right)\\ \;§O_{2}+2\;e^{-}+H_{2}O=2{\mathrm{OH}}^{-}\\ \;2{\mathrm{OH}}^{-}+2h^{+}\;=2{\mathrm{OH}}^{.}\\ {\mathrm{CN}}^{-}+2\;{\mathrm{OH}}^{.}={\mathrm{OCN}}^{-}+H_{2}O\\ \;2{\mathrm{OCN}}^{-}+O_{2}=2{\mathrm{CO}}_{2}+N_{2} \end{array}$$

The overall reaction:

$$2{\mathrm{CN}}^{-}+2O_{2}\;\overset{\mathrm{ZnO}/H_{2}O}{\underset{\mathrm{UV}\;\mathrm{light}}{\to }}2{\mathrm{CO}}_{2}+N_{2},$$

where *h* is Planck's constant and *ν* is the frequency of UV light.

The effect of the synthesis medium on the photocatalytic efficiency of calcined ZnO nanoparticles was explicitly noticed by the much higher efficiency of ZnO_E_ than that of ZnO_W_ in the photocatalytic degradation of cyanide ion in the aqueous medium under the same conditions. Table 4 shows that the photocatalytic activity of ZnO_E_ is as approximately 1.5 as that of ZnO_W_ when applying 0.02 wt.% concentration of the ZnO photocatalyst. The higher performance of ZnO_E_ can be attributed to the higher adsorption capability of its particles, owing to its regular, polyhedral surface faces.

**Table 4:** Effect of the synthesis medium on photocatalytic activity

**Sample**	**ZnO loading (wt.%)**	**CN‾ degradation (%)**
ZnO_E_	0.02	86
ZnO_W_	0.02	56

The superiority of ZnO_E_ photocatalytic activity can be correlated to its particle size and shape, as it is reported in the literature [42-45]. However, the effect of ZnO particle shape on the photocatalytic activity is rarely studied in the literature [46]. In this context, the edges and corners of ZnO_E_ hexagonal particles have many coordinatively unsaturated sites, which usually are active in catalysis. On the other hand, the spherical shape of ZnO_W_ particles would have much less active sites due to the lack of edges and corners. Aligning with our interpretation of ZnO_E_ photocatalytic activity, El-sayed and his coworkers, for instance, showed that the influence of the particle shape on the catalytic activity is very important toward better activity [42,45]. In addition, the photocatalytic activity of acetaldehyde decomposition using ZnO powder depended on several factors including the morphology of the particles [46]. Finally, we believe that the morphology of our ZnO_E_ particles is crucial in photocatalytic activity and our present findings will provide a hint about the role of morphology in the ZnO_E_ photocatalytic performance.

Based on the obtained results, ZnO_E_ nanoparticles were used in further investigation for improving the cyanide degradation efficiency.

### Photocatalytic degradation of CN^-^ using different concentrations wt.% of calcined ZnO_E_

**Figure 7:**
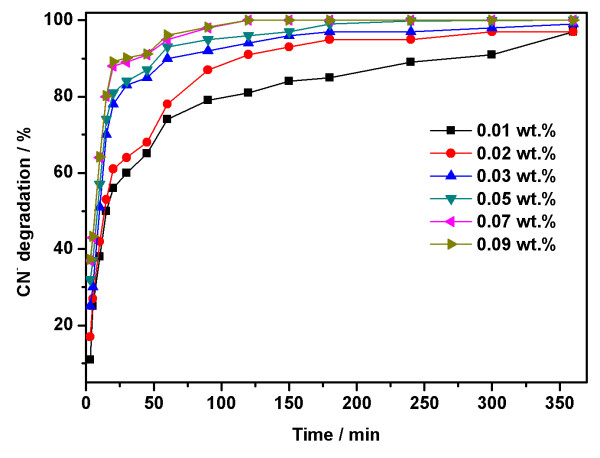
**Photocatalytic degradation of cyanide using different concentration wt.% of calcined ZnO**_**E**_**.** Reaction conditions: 100 ppm KCN_(aq)_, *t* = 25°C, pH = 8.5.

Photocatalytic degradation of cyanide using different weight percent of calcined ZnO_E_ was performed and found to depend on the ZnO concentration wt.%, as shown in Figure 7. It is evident that at the initial reaction stage, the catalyst concentration of ZnO has no notable effect on the catalytic performance, which might due to the high essential activity of the ZnO_E_ catalyst. It is clear from Figure 6 that the smallest concentration of 0.01 wt.% ZnO_E_ resulted in cyanide degradation of 85% after 180 min, while it increased remarkably to 95% with increasing the loading from 0.01 to 0.02 wt.%. However, further increase in the ZnO_E_ concentration from 0.02 to 0.09 wt.% had resulted in almost 100% CN removal efficiency. This observation might be due to the increase in photon absorption by the ZnO_E_, resulting in higher concentration of the charge carrier to degrade almost all CN^-^_(aq)_. The degradation of cyanide, however, remained relatively constant with further increase in the reaction time beyond 180 min, indicating that the catalyst might be deactivated by deposition of the reaction products on the catalyst surface.

### Kinetic photocatalytic degradation of CN^-^ using calcined ZnO_E_

**Figure 8:**
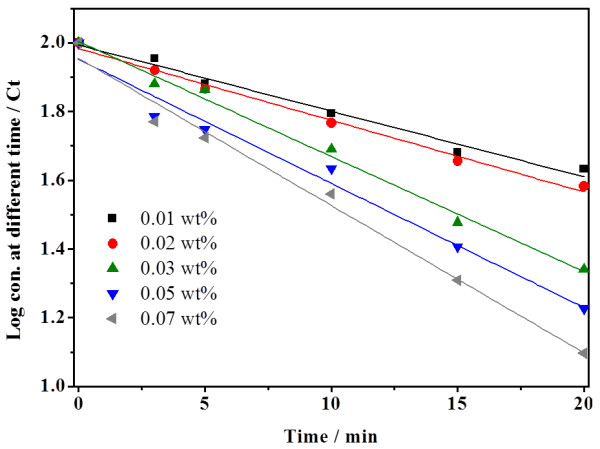
**Photodegradation kinetic of cyanide ion over calcined ZnO**_**E**_**.**

The first order kinetic degradation of CN^**–**^_(aq)_ was fitted to the following expression:

$$\mathrm{Log}{\left[C\right]}_{t}=-\mathrm{kt}+\mathrm{Log}{\left[C\right]}_{o},$$

where [*C*]_t_ and [*C*]_o_ represent the concentration in (ppm) of CN^¯^_(aq)_ in solution at time zero and at time *t* of illumination, respectively, and *k* represents the apparent rate constant (min^-1^). The kinetic analysis of cyanide photodegradation is depicted in Figure 8, which shows that the rate of photocatalytic reaction depends on the concentration of the catalyst. An excellent correlation to the pseudo-first-order reaction kinetics (*R* > 0.99) was found. Obviously, the photodegradation rate of the CN^-^ was found to increase from 19.2 to 42.9 × 10^-3^ min^-1^ with increasing ZnO loading from 0.01 to 0.07 wt.% (Table 5).

**Table 5:** **Apparent rate constant (*k*) at different concentration wt.% of calcined ZnO**_**E**_

**ZnO**_**E**_**concentration, wt.%**	***k*** **(min × 10**^**-3**^**)**
0.01	19.2
0.02	20.8
0.03	33.5
0.05	36.1
0.07	42.9

## Conclusion

Zinc oxide nanoparticles were readily prepared at room temperature from zinc nitrate hexahydrate and cyclohexylamine either in aqueous or ethanolic medium. The calcined ZnO_E_ had a regular, polyhedra morphology while the calcined ZnO_W_ had irregular spherical morphology, mixed with some chunky particles. The morphology was a key factor in the superior photocatalytic behavior of ZnO_E_ over that of ZnO_W_. The differences in morphology and photocatalytic behavior are strongly influenced by the physicochemical properties of the synthesis medium.

## Competing interests

The authors declare that they have no competing interests.

## Authors' contributions

All authors have contributed to the final manuscript of the present investigation. AB and AA have defined the research topic, the preparation, the characterization, and photocatalytic experiments. AB, AA, and MA wrote the manuscript. HK provided important suggestions on the draft manuscript. All authors examined and approved the final manuscript.
